# Neural Correlates for Apathy: Frontal-Prefrontal and Parietal Cortical- Subcortical Circuits

**DOI:** 10.3389/fnagi.2016.00289

**Published:** 2016-12-09

**Authors:** Rita Moretti, Riccardo Signori

**Affiliations:** Neurology Clinic, Department of Medicine, Surgery and Health Sciences, University of TriesteTrieste, Italy

**Keywords:** apathy, frontal cortex, striatum, parietal lobe, nucleus accumbens, anterior cingulate cortex

## Abstract

Apathy is an uncertain nosographical entity, which includes reduced motivation, abulia, decreased empathy, and lack of emotional involvement; it is an important and heavy-burden clinical condition which strongly impacts in everyday life events, affects the common daily living abilities, reduced the inner goal directed behavior, and gives the heaviest burden on caregivers. Is a quite common comorbidity of many neurological disease, However, there is no definite consensus on the role of apathy in clinical practice, no definite data on anatomical circuits involved in its development, and no definite instrument to detect it at bedside. As a general observation, the occurrence of apathy is connected to damage of prefrontal cortex (PFC) and basal ganglia; “emotional affective” apathy may be related to the orbitomedial PFC and ventral striatum; “cognitive apathy” may be associated with dysfunction of lateral PFC and dorsal caudate nuclei; deficit of “autoactivation” may be due to bilateral lesions of the internal portion of globus pallidus, bilateral paramedian thalamic lesions, or the dorsomedial portion of PFC. On the other hand, apathy severity has been connected to neurofibrillary tangles density in the anterior cingulate gyrus and to gray matter atrophy in the anterior cingulate (ACC) and in the left medial frontal cortex, confirmed by functional imaging studies. These neural networks are linked to projects, judjing and planning, execution and selection common actions, and through the basolateral amygdala and nucleus accumbens projects to the frontostriatal and to the dorsolateral prefrontal cortex. Therefore, an alteration of these circuitry caused a lack of insight, a reduction of decision-making strategies, and a reduced speedness in action decision, major responsible for apathy. Emergent role concerns also the parietal cortex, with its direct action motivation control. We will discuss the importance of these circuits in different pathologies, degenerative or vascular, acute or chronic.

## Introduction

The clinical importance of apathy is more and more amplified in neuro-pathological context, especially and at the moment, related specifically to frontal-subcortical circuit alteration (Starkstein et al., 1992; Duffy, 2000; Aarsland et al., 2001; Starkstein and Leentjens, 2008; Massimo et al., 2009; Chase, 2011; Moretti et al., 2012). The overt definition of apathy is that of a reduced motivation, which directly involve the goal-directed behavior (Duffy, 2000) with a diminishment of emotional involvement, and difficulty in the beginning of new actions (Duffy, 2000).

Apathy is a widespread condition, in many neurological pathology, whose recognition might be helpful for dedicated therapy (Hoehn-Saric et al., 1990; McConnell et al., 1996; Campbell and Duffy, 1997; Castellon et al., 1998; Kant et al., 1998; Diaz-Olavarrieta et al., 1999; Okada et al., 1999; Cummings, 2000).

If not entirely accepted, there are many different categorization of apathy; one of the most anatomical and patho-physiological is the one, proposed (Duffy, 2000) in which different anatomical networks are involved: therefore, Duffy (2000) proposed the terms of cognitive apathy, as the one caused by an alteration of the dorso-frontal cortex, and which brings to dyseexecutive alteration; of motor apathy (Duffy, 2000) with an alteration of motor speedness and execution, mainly related to the extra-pyramidal disrupted circuits(Duffy, 2000); of cortical-sensory apathy (Duffy, 2000), due to altered teleceptive process, and therefore causing the inability to motivate and act as a consequence of sensory stimuli; and of emotional apathy(Duffy, 2000), due to an incapacity to employ the inner planning and voluntary stimulus to act with a defined aim, for a disorder of the extended region of the amygdala (Marin, 1997a,b; Duffy, 2000).

Robert et al. (2009) proposed novel diagnostic criteria for apathy which have been most widely used in recent studies. These can be summarized as lack of motivation associated with lack of:

1. Goal-directed behavior;
2. Goal-directed cognitive activity;
3. Spontaneous or reactive emotional expression, frequently characterized as “emotional blunting.”

In more recent days, Quaranta et al. (2012) connected the occurrence of apathy to damage of prefrontal cortex (PFC) and basal ganglia (Chase, 2011), and define it as:

1. Emotional apathy (associated with damages of ventral striatum and orbitomedial PFC behavior form of FTD bvFTD; Quaranta et al., 2012);
2. Cognitive apathy (correlated with an impairment of the lateral PFC and dorsal caudate nuclei; Quaranta et al., 2012);
3. Deficit of auto-activation (Quaranta et al., 2012; derived from the bilateral lesions of the dorsomedial portion of PFC, the internal portion of globus pallidus, or bilateral paramedian thalamic lesions; Marin et al., 1991; Marin, 1996; Levy and Dubois, 2006; Peters et al., 2006; Zamboni et al., 2008; Chow et al., 2009; Moll et al., 2011).

As far as we have above reported, there are many different possible systematic categorization for apathy, but, at the moment, there is not an adequate and world- accepted gold-standard scale to measure apathy; in particular, there are many shortcomings of the different existing scale, due to the fact that some are heavily standing on caregivers, some other consider apathy in the context of different other neuropsychiatric events (Loewenstein et al., 2001; Njomboro and Deb, 2012; Cummings et al., 2015a,b). What it is strongly recommended is that the clinician should evaluate apathy inside the clinical behavior and cognitive status of the patient, and even in his environment situation (Duffy, 2000).

### What do we know from animal experiments and single case studies

The frontal-subcortical circuitry provides a unifying framework for understanding apathy (Alexander, 1994; Litvan, 2001; Monchi et al., 2006; Bonelli and Cummings, 2007).

Alexander et al. (Alexander et al., 1986, 1990; Alexander and Crutcher, 1990; Alexander, 1994) proposed that the basal ganglia and thalamus are intimately related to frontal cortex and the inside of subcortical-frontal networks, in five parallel circuits (Alexander et al., 1986, 1990; Alexander and Crutcher, 1990; Alexander, 1994). Two of these circuits influence the skeletal motor executive function and the ocular-motor areas.

The other three loops connect the basal ganglia and thalamus to the dorsolateral prefrontal cortex the lateral orbitofrontal cortex and the anterior cingulate/medial orbitofrontal areas (Alexander et al., 1986, 1990; Alexander and Crutcher, 1990; Alexander, 1994). The areas are involved in executive functions, attention, working-memory, focusing attention, and judgment (Fielding et al., 2006; Bonelli and Cummings, 2008). PET and fMRI imaging publications confirmed *in vivo* these models (Postuma and Dagher, 2006; Bonelli and Cummings, 2007).

The topical organization of the prefrontal cortex network is strongly reflected in the dynamic development of prefrontostriatal regions of connections (Yeterian and Van Hoesen, 1978; Yeterian and Pandya, 1991; Flaherty and Graybiel, 1993; Lattery et al., 2001; Bonelli and Cummings, 2007). The striatum recombined the information derived from the cortex to form small and specialized areas (Divac et al., 1967; Johnson et al., 1968; Divac, 1972; Brown, 1992; Lichter and Cummings, 2001; Hirata et al., 2006).

The two motor circuits (Lehericy et al., 2006) send impulses directly toward the putamen, then to ventrolateral globus pallidus internal (GPi), globus pallidus external (GPe), and caudolateral SN. The globus pallidus (GP) connects to the ventrolateral, ventral anterior, and centromedianum nuclei of the thalamus, which send projections directly to the supplementary motor area, premotor cortex, and motor cortex, completing this way the circuit.

There are three other circuits, which starts from BG and arrives to frontal areas, which are behaviorally relevant:

A dorsolateral-prefrontal circuit, which seems involved in executive functions (Alexander and Crutcher, 1990; Alexander et al., 1990; Alexander, 1994);

An anterior cingulate circuit, involved in motivational mechanisms (Alexander and Crutcher, 1990; Alexander et al., 1990; Alexander, 1994);

An orbitofrontal circuit, with lateral and medial sections. The medial part permits integration of visceral-amygdalar functions with the internal state of the organism; the lateral portion is involved with transformation of limbic and emotional information into contextually appropriate behavioral responses (Alexander and Crutcher, 1990; Alexander et al., 1990; Alexander, 1994).

Each circuit has two pathways:

1. Direct pathway, featuring a monosynaptic link between the GPi-SN pars reticulata (SNr) complex;
2. Indirect pathway, projects from striatum to GPe, connecting to the Gpi-SNr complex via the subthalamic nucleus (STN; Alexander and Crutcher, 1990; Alexander et al., 1990).

Both direct and indirect circuits project to the thalamus.

Although, each frontal-subcortical circuit constitutes a closed loop of anatomically segregated dedicated neurons, “open”-loop elements are incorporated into their functional connectivity, including parietal cortex, thalamic nuclei, prestriate cortex, and amygdala nuclei (Groenewegen and Berendse, 1990; Parent, 1990; Weinberger, 1993; Salmon et al., 2001; Bonelli et al., 2006; Bonelli and Cummings, 2007).

The dorsolateral prefrontal circuit begins in the cortical regions of areas 9 and 10, projects to the caudate nucleus (Selemon and Goldman-Rakic, 1985), via the direct pathways to the lateral faces of the GPi and SNr (Parent et al., 1984). The indirect pathway project to the dorsal GPe, and subsequently to the lateral STN (Smith and Bolam, 1990). Neurons from the lateral STN send afferents to the GPi-SNr complex. The output efferents from the basal ganglia project directly to the mediodorsal and ventral anterior thalamus (Kim et al., 1976; Illinsky et al., 1985), and return to Brodmann's areas 9 and 10 (Kievit and Kuypers, 1977; Giguere and Goldman-Rakic, 1988), relating these circuits to executive function. The prefrontal cortex can be considered as the most developed cortical site for the control and the correct operative selection of executive functions and appropriate behavior (Kolb et al., 2004; Nácher et al., 2006; Alexander and Brown, 2011). As pointed out by Leal-Campanario et al. (2013) it seems more precise to better define the prefrontal cortex into the dorsolateral part, which seems to be tightly bound to the working memory and to the correct operative choice of appropriate behavior, and the medial prefrontal area, which appears to guarantee the emotional color of intentional action and of the acquired act, including for animals and humans, as perfectly stated by Leal-Campanario et al. (2013) “associative conditioning” (Weiss and Disterhoft, 2011; Jurado-Parras et al., 2012). Studies based on transcranial magnetic stimulation support the hypothesized role of the prefrontal cortex (Petrosini, 2007) and experimental data extend it toward its projection on nucleus accumbens (Jurado-Parras et al., 2012) in imitative learning in humans. To become even more precise, the caudal medial prefrontal part participate in the gaining, and in the recovery of the conditioning (Simon et al., 2005), whereas the rostral medial part restrains the conditioned act sequence, without limiting its acquisition (Leal-Campanario et al., 2007, 2013). To the rostral part of the medial dorsal prefrontal cortex widely projects the mediodorsal thalamic nucleus (Leal-Campanario et al., 2007, 2013) and diffuse projections from it and arrives to the anterior cingulate cortex (ACC), exerting on it an inhibitory effect, but also suggesting that the effects of this area is not constant and univocal, but it acts as an ongoing control system, decreasing or increasing the operative execution of a conditioned response, in relation to environment, time, and occurrence. In fact, what clearly merge from another important study (the rostral medial prefrontal cortex inhibits the execution of the reflexed motor act, evoking what Leal-Campanario et al., 2007) defined as “a freezing behavior,” but rather curiously, this marked inhibition to-act system does not reduce the possibility to acquire different conditioned response. On the contrary, when there is a cortical depression of this area, the animal can act more rapidly in response to a condition stimulus (Weiss and Disterhoft, 2011; Leal-Campanario et al., 2013).

This ongoing control process is exerted directly from the discharge of the rostral medial part of prefrontal cortex, but highly supported by its diffuse projections to caudate, and claustrum and via these nuclei to the basal ganglia, mediodorsal thalamic nuclei, as above mentioned, and to midline thalamic nuclei, highly related to the process of arousal, selective attention, but also to the substantia nigra pars reticulate, fundamental for motor action incipit (Basso and Evinger, 1996; Kronforst-Collins and Disterhoft, 1998; Siegel et al., 2012; Leal-Campanario et al., 2013), to the superior colliculus and to pontine nuclei (Kronforst-Collins and Disterhoft, 1998; Fuster, 2001; Siegel et al., 2012).

The orbitofrontal circuit begins in the lateral orbital gyrus of area 11 (Mega and Cohenour, 1997; Mega et al., 1997) and in the medial inferior frontal gyrus of the areas 10 and 47 in humans (Mega and Cohenour, 1997; Mega et al., 1997), send projections to caudate, then to the SNr and to GPi (Johnson and Rosvold, 1971). The caudate is the start point of an indirect loop (Smith et al., 1990), which passes through the dorsal GPe, the STN side, and reaches GPi and SNr (Smith et al., 1990). Neural networks from the GP and SN to the mediodorsal thalamus and ventral anterior thalamus (Illinsky et al., 1985; Selemon and Goldman-Rakic, 1985) complete the neural loop into the lateral orbitofrontal cortex (Illinsky et al., 1985).

There is another division of this circuit that has been identified by various studies (Mega and Cohenour, 1997; Mega et al., 1997): the fibers originate from inferomedial prefrontal cortex, and the medial orbital gyrus of area 11 (Mega and Cohenour, 1997; Mega et al., 1997) and project to the accumbens, to medio-ventral pallidum; they arrive into the mediodorsal thalamic nucleus, and to conclude the loop arrive in the medial orbitofrontal cortex (Mega and Cohenour, 1997; Mega et al., 1997). This portion of the cortex has reciprocal relations with the magnocellular division of the accessory basal amygdala (Mega and Cohenour, 1997; Mega et al., 1997). The orbitofrontal inferomedial cortex has many other ways of connecting with the rostral insula, and Brodmann's areas 24, 25, 32 (infracallosal cingulate areas), and 38, regions that are part of the ACC (Johnson and Rosvold, 1971; Mega and Cohenour, 1997; Mega et al., 1997). The orbitofrontal cortex receives inputs from the limbic system (Lichter and Cummings, 2001) and participate in the awareness, in the insight, in the appropriate, and predefinite social conduct (Eslinger and Damasio, 1985; Starkstein and Kremer, 2001). In fact, ACC is the trait d'union between area 24 and the ventromedial caudate, putamen, nucleus accumbens, and olfactory tubercle (Selemon and Goldman-Rakic, 1985), originally defined as the limbic striatum (Levy and Dubois, 2006); from these nuclei the fibers arrives to the GPi and GPe(Lichter and Cummings, 2001) and to SN (Critchley, 2005). Now, the connection system, at this point, departs from GPe and arrives to STN, which directs to the ventral pallidum (Leal-Campanario et al., 2013), from that point via the mediodorsal thalamus (Critchley, 2005) and conclude the loop into the anterior ACC (Goldman-Rakic and Porrino, 1985).

Two clinical conditions have been related to lesions in these regions.

The first one is the “Akinetic mutism,” clinically related to a lesion of the anterior cingulate, either unilaterally, either bilaterally (Ackermann and Ziegler, 1995; Mega and Cohenour, 1997; Mega et al., 1997; Oberndorfer et al., 2002). Akinetic mutism is a dramatic clinical condition where the human being is apathic, totally deprived of motivation, absent of primary stimuli, such as hunger, psycho-motor initiative, lack of verbalization, and inability to answer questions or commands (Goldman-Rakic and Porrino, 1985; Ackermann and Ziegler, 1995; Mega and Cohenour, 1997; Mega et al., 1997; Oomman and Madhusudhanan, 1999; Anderson et al., 2003; Tengvar et al., 2004).

The second one is apathy, driven out by bilateral lesions of ventrolateral and dorsomedial thalamic nuclei (Bogousslavsky et al., 1988; Levy and Dubois, 2006), GP and the internal capsule (Helgason et al., 1988; Starkstein et al., 1993), the ansa lenticularis (internal pallidal, posterior limb of the internal capsule, and end in the peduncolopontine nucleus; Bechara and van der Kooy, 1989; Bhatia and Marsden, 1994; Ackermann and Ziegler, 1995). We can induce a syndrome similar to akinetic mutism injecting 6-hydroxy dopamine in the SN, ventral tegmental area, or nigrostriatal tract within the medial forebrain bundles of the lateral hypothalamus (Ungerstedt, 1970, 1971). Administration of apomorphine (a direct dopamine agonist) could reverse behavioral deficits (Ungerstedt, 1971; Marshall and Ungerstedt, 1976) and a pretreatment with spiroperidol (a dopamine receptor antagonist), could block them (Marshall and Gotthelf, 1979).

Confirmation of these observations came from the case of a patient who developed akinetic mutism due to surgical removal of a tumor of the anterior hypothalamus who responded to treatment with lergotrile and bromocriptine (dopamine receptor agonists), but not to carbidopa/L -dopa or methylphenidate (presynaptic dopamine mimetics; Ross and Stewart, 1981). This case suggested that the base of akinesia and apathy lies on a direct loss of dopaminergic input to the cingulate or other corticolimbic structures rather than to the striatum (Nemeth et al., 1986; Echiverri et al., 1988; Combarros et al., 2000; Alexander, 2001).

As with akinetic mutism, even for apathy, it was observed a substantial response to treatment with dopamine-agonists, suggesting a common role of dopaminergic pathways in both conditions (Marin et al., 1995; Watanabe et al., 1995; Lichter and Cummings, 2001). Apathy that appears in experimental models of Alzheimer's disease can be also interpreted as an alteration of cholinergic disconnections of structures of the ACC, e.g., basal nucleus of the amygdala (Mega and Cohenour, 1997; Mega et al., 1997), and the paramedian thalamic portions, probably for their intrinsic role of connecting basal forebrain to the ARAS system, deriving from the cholinergic pedunculo-pontine projections.

To summarize, we can detect a theoretical model for the Neural Substrates of Motivation traced out by Kalivas: (Kalivas et al., 1994)

*Subcircuit number 1* (Kalivas et al., 1994): this circuit is mediated by a loop via the ventral tegmentum, through the nucleus accumbens and to the ventral pallidum. As Kalivas pointed out, this circuit gives motivation to the operative process, in the “motivational working memory” (Kalivas et al., 1994).

*Subcircuit number 2* (Kalivas et al., 1994): this circuit is composed by the ventral pallidum, the medial dorsal nucleus of the thalamus, the prefrontal cortex, the nucleus accumbens, and the ventral tegmentum. As Kalivas (Kalivas et al., 1994) underlined this network provides “the cognitive coloring of motivation” (Kalivas et al., 1994).

*Subcircuit number 3* (Kalivas et al., 1994): this circuit is composed by the projections via the ventral pallidum, the pedunculo-pontine nucleus and the ventral tegmentum, and strengths the passage from the arousal into motivation (Kalivas et al., 1994).

*Subcircuit number 4* (Kalivas et al., 1994): this circuit occurs between the ventral tegmentum and nucleus accumbens, via the amygdala and implements the “reward memory into motivational response” (Kalivas et al., 1994).

Dopamine is the principal neurotransmitter of the four dopaminergic systems; nigro-striatal, mescortical, meso-limbic, and tubero-infundibular vias. Dopamine subserves various system, in particular concerning the arousal, the motor refinement system, the goal-motivation (Duffy, 1997a,b, 2000).

The cholinergic network is a widespread system, which originates form the Meynert nucleus, participate to the ARAS system, which arrives to the mesencephalic and tectal region, the limbic system, regulates the extrapyramidal nigro-striatal dopaminergic system, the thalamic nuclei, and basal forebrain. Therefore, cholinesterase and butyrrilcholinesterase inhibitors have been employed for apathy therapy (Hoehn-Saric et al., 1990; Duffy, 2000).

Serotonin, a part from the mood and pain modulation regulatory system, via raphe magnum and frontal projections, has an intrinsic activity on enhancing the dopamine networks of the ventral tegmentum and nucleus accumbens (Duffy, 2000) via the 5-HT3 receptors, reinforcing therefore motivational tenor (Kalivas et al., 1994; Duffy, 2000) (Supplementary Material).

## What do we know about apathy from clinical practice?

Apathy is common in distinct neurological disorders. There are no authoritative estimates available on the prevalence of apathy in general, but it has been estimated that ~10 million people in the US suffer from apathy (Chase, 2011; Clarke et al., 2011; van Dalen et al., 2013). Across various disorders, apathy is regarded as the strongest predictor of poor cognitive, functional, and occupational outcome, reduced medication compliance, increased caregiver burden, diminished quality of life, and general health (Levy et al., 1998; Stuss et al., 2000; van Reekum et al., 2005; Guimaraes et al., 2008; Dujardin et al., 2009; Ishii et al., 2009; Starkstein et al., 2009; Jorge et al., 2010; Benoit and Robert, 2011; Chase, 2011; Clarke et al., 2011; Kostic and Filippi, 2011; Hsieh et al., 2012; Caeiro et al., 2013; Moretti et al., 2013; Santangelo et al., 2013; van Dalen et al., 2013; Stella et al., 2014; Theleritis et al., 2014; Fervaha et al., 2015; McIntosh et al., 2015).

### Apathy following cerebrovascular accidents

Many data seem to forewarn the clinician that a patient with CVS may develop an apathy syndrome (which has been defined as a Post-Stroke-Apathy, PSA; Brown and Pluck, 2000). Tatemichi et al. (1992) postulate that stroke lesions to the posterior limbs of internal capsule disrupt the major outflow tract of the internal pallidum, the ansa lenticularis, which connects to the mesencephalic locomotor region (MLR), critical in generating goal-oriented behavior (Tatemichi et al., 1992; Kos et al., 2016). Okada et al. (1997) reported that apathy is related to reduced cerebral blood flow in the right dorsolateral frontal and left frontotemporal regions (Duffy, 2000; Hama et al., 2007).

Stroke patients with apathy showed more right-sided lesions in general, and specifically more white- matter hyperintensities within the right fronto-subcortical circuit (Okada et al., 1997; Brodaty et al., 2005; Caeiro et al., 2012), to higher amount of perventricular white matter hyperintensities (Finset and Andersson, 2000), lower fractional anisotropy values (FA) in the anterior corona radiata and right inferior frontal gyrus (Tang et al., 2013), and an increased number of microbleeds (Yang et al., 2015). In a DTI connectivity analysis in stroke patients by Yang et al. (2015) an “apathy-related sub-network” merged in relation with apathy: the right supra-marginal gyrus, right precuneus, and right paracentral lobule, the right superior temporal gyrus, bilateral insula (Yang et al., 2015), the hippocampus, right putamen, right thalamus, and posterior cingulum (Yang et al., 2015).

Within a study focusing apathy due to subcortical lesions, regional changes at a distal location from the lesion site were reported, namely in the posterior cingulate cortex (Tatemichi et al., 1992; Deguchi et al., 2013; Matsuoka et al., 2015), bilateral basal ganglia damage (Levy et al., 1998; Moretti et al., 2013), isolated pontine or cerebellar infarcts (Piamarta et al., 2004; Hoffmann and Cases, 2008; Onoda et al., 2011).

### Alzheimer's disease

Alzheimer's disease (AD) affects memory and cognition, but all AD patients develop neuropsychiatric symptoms (NPS; Lyketsos et al., 2000, 2002; Steinberg et al., 2008; Onoda and Yamaguchi, 2011), including apathy (Marin et al., 1991; Robert et al., 2009). NPS is the most important cause of increase of caregiver'stress, but there is no general concordance or FDA-approved medications for NPS in AD (Sultzer et al., 2008; Rosenberg et al., 2013; Porsteinsson et al., 2014; Peters et al., 2015).

An important study by Benoit et al. (1999) demonstrated that apathy but not depressed patients had significantly lower scores on cognitive tests (Porsteinsson et al., 2014).

Ample epidemiological data indicate, on the other hand, that in AD, individual NPS rarely occur alone (Hoffmann and Cases, 2008). While, these groupings have been supported by limited studies (Jonsson et al., 2010; Geda et al., 2013) others do not support this approach (Jeste and Finkel, 2000; Olin et al., 2002; Robert et al., 2009; Trzepacz et al., 2013; Cummings et al., 2015a,b). Data, therefore, might be confused and not unequivocal at all. Many data established a possible link between apathy and AD with a decreased reliability of ACC network, mainly due to an increased generalized amyloid depositions (Canevelli et al., 2013), a volume loss in ACC (Apostolova et al., 2007; Bruen et al., 2008; Marshall et al., 2013; Stanton et al., 2013), a decreased perfusion in ACC (Benoit et al., 2004; Lanctôt et al., 2007; Tunnard et al., 2011), a decreased ACC white matter related integrity (Robert et al., 2006; Kim et al., 2011; Hahn et al., 2013), and increased amyloid burden in right ACC (Ota et al., 2012; Mori et al., 2014). Apathy is also associated with decreased posterior cingulate (PCC metabolism; Migneco et al., 2001), with a reduced insular volume (Moon et al., 2014a; Delrieu et al., 2015), and with decreased inferior temporal cortical (ITC) thickness (Moon et al., 2014b; Guercio et al., 2015), with cortical shrinkage of the frontal cortex (Apostolova et al., 2007; Bruen et al., 2008; Marshall et al., 2013; Stanton et al., 2013), with greater amyloid burden in bilateral frontal cortex (Ota et al., 2012), reduced orbitofrontal metabolism on the left (Donovan et al., 2014) or right (Benoit et al., 2004), and reduced connectivity in left-sided functional connectivity, with thalamus and parietal cortex, and amygdala (Holthoff et al., 2005; Kang et al., 2012; Ota et al., 2012; Baggio et al., 2015). Galantamine, as a cholinesterase inhibitor has been linked to a slower decrease of the putamen metabolism (Zhao et al., 2014), based on FDG-PET study (Zhao et al., 2014). One neurochemical study of CSF in apathy-patients suffering from AD (Mega et al., 2005) demonstrated an higher levels of phospho-tau, related to advanced neurdegenerative process (Skogseth et al., 2008).

Using 99 m-Technetium SPECT cerebral blood flow (CBF) abnormalities were found in bilateral frontal regions: negative correlations were found between apathy and orbitofrontal regions (Benoit et al., 2004; Skogseth et al., 2008), right inferior frontal (Jack et al., 2013), and the right and bilateral medial (Jack et al., 2013; Baggio et al., 2015), and dorsolateral regions, right lingual gyrus (Craig et al., 1996; Benoit et al., 2002; Robert et al., 2006), right posterior temporo-parietal area (Schroeter et al., 2011).

Mori et al. (2014) investigated amyloid-B (AB) deposition using ((11)C) Pittsburgh Compound-B (PiB) PET in relation to apathy in AD: elevated levels of AB are seen (Mori et al., 2014) throughout the whole frontal cortex, bilateral insula, and right ACC.

AD patients white matter alterations related to apathy have been investigated (Kostic and Filippi, 2011). Often DTI is used to evaluate white matter integrity, as quantified with fractional anisotropy (FA). In patients with high apathy, lower FA values were found within the left, right, or bilateral anterior and posterior cingulum, the genu, body, and splenium of the corpus callosum (Holthoff et al., 2005; Hahn et al., 2013; Baggio et al., 2015).

Furthermore, diffusion abnormalities in the right thalamus and parietal regions were associated with higher apathy (Mori et al., 2014; Kang et al., 2012). Data are not univocal (Donovan et al., 2014; Delrieu et al., 2015). One fMRI study found alterations in functional limbic-hypothalamic networks in relation to apathy (Tatemichi et al., 1992; Starkstein et al., 1997; Balthazar et al., 2014).

Furthermore, one study that included both AD and Lewy body disease patients, investigated the relationship between apathy and striatal dopamine uptake, using (123)I-FP-CIT SPECT (Aalten et al., 2008): higher apathy was associated with a reduced dopaminergic binding potential in the right putamen (Aalten et al., 2008). On the other hand, another study reported no association with D2/D3 dopamine receptor density (David et al., 2008; Reeves et al., 2009; Cuthbert and Insel, 2013; Grupe and Nitschke, 2013; Rosenberg et al., 2013).

In our opinion, two of the most significant studies which start form the observation that AD patients did not frequently show isolated NPS symptoms, and that overlap networks should be taken into account are below reported.

Rosenberg et al. (2013) hypothesized, that apathy is one of the many different coexistent behavior symptoms in AD, and therefore these symptoms correlate with a dysfunction in several structures such as ACC, orbitofrontal cortex, and insula, as well as amygdala and striatum. As stated by Grupe and Nitschke (2013), apathy might be the behavior response toward the anxiety-provoking situations, which might be very normal and daily living activity, but inducing apprehension in AD patients (Grupe and Nitschke, 2013). Therefore, in AD one can observe an altered balance mechanism, which alterantively regulates apathy or anxiety.

### Parkinson's disease

The very first study on apathy and neurodegenerative pathology concerns Parkinson's Disease (PD): Starkstein et al. (1992) reported that in PD patients apathy is a serious problem, related to greater cognitive impairment, and to depletion of catecholamines in the locus ceruleus. Another clinical study reported that apathy tended to cluster with anxiety, whereas hallucinations, delusions, and irritability formed another distinct behavioral cluster (Insel et al., 2010). There are not adequate existamated prevalence of apathy in PD, arising up to 51% of the entire PD population (Duffy, 2000; Stuss et al., 2000; Starkstein et al., 2009), depending on the assessment and also deriving form not universally considered inclusion/exclusion criteria (Levy et al., 1998; Stuss et al., 2000; Starkstein and Leentjens, 2008; Starkstein et al., 2009; Moretti et al., 2013; many studies included patients with super-imposition of AD with parkinsonism (Stuss et al., 2000; Starkstein and Leentjens, 2008; Dujardin et al., 2009; Starkstein et al., 2009). Starting from the anatomical consideration of dopaminergic pathways, all the four dopaminergic mainstreams, the nigro-striatal, the meso-limbic, the meso-cortical, and the tubero-infundibular dopaminerg can somehow been involved in apathy determination in PD patients (Ljungberg and Ungerstedt, 1976; Insel et al., 2010; Cuthbert and Insel, 2013). But apathy in PD does not seem dependent on the duration of the disease, the severity of symptoms and the dosage of Levodopa; everything indicates that the brain changes that lead to apathy in Parkinson's disease are different from those involved in the apathy associated with motor symptoms (Levy et al., 1998; Dujardin et al., 2009; Moretti et al., 2013). Apathy and depression are quite divided symptoms, with a prevalence of depression than apathy in progressive supranuclear palsy (Insel et al., 2010; Cuthbert and Insel, 2013) of apathy in corticobasal degeneration (Levy et al., 1998; Litvan et al., 1998; Aarsland et al., 1999; Moretti et al., 2013).

Considering the specific information deriving from neuroimaging, apathy in PD has been studied (Tatemichi et al., 1992).

Reijnders et al. (2010) found an association between higher apathy and lower gray matter density values with voxel based morphometry analyses, in the bilateral inferior parietal gyrus, and right precuneus (Reijnders et al., 2010). Skidmore et al. (2013) investigated functional integrity of the brain in relation to apathy by using voxel-wise fractional amplitude of low frequency fluctuations analysis in resting state fMRI data: apathy was best predicted by a lower signal amplitude in the right middle orbitofrontal cortex and bilateral subgenual cingulate cortex, in the left supplementary motor cortex (SMA), the left inferior parietal lobule, and left fusiforme gyrus (Robert et al., 2012; Skidmore et al., 2013). According to the review by Kos et al. (2016) multiple fluorodeoxyglucose (FDG-) PET-studies specifically found a positive correlation of apathy and cerebral metabolism during rest in the right middle frontal gyrus, right inferior frontal gyrus, left anterior insula (Skidmore et al., 2013), bilateral orbitofrontal lobes, and bilateral anterior cingulate (Robert et al., 2012), and left posterior cingulate cortex (Huang et al., 2013), an increased cerebral metabolism in the right cuneus (Skidmore et al., 2013) and reduced metabolism within the right inferior parietal lobe and left superior temporal gyrus (Robert et al., 2012). Functional connectivity within the striatum and between striatal and ventrolateral prefrontal regions was more impaired in patients with high apathy compared to low (Holthoff et al., 2005). Data are not univocal, since two other studies (Robert et al., 2009, 2014) did not find out any structural differences when comparing apathetic to non-apathetic PD patients.

As strongly pointed out by Kos et al. (2016), it has been found an inverse correlation between catecholaminergic binding potential, indicative of a specific loss of dopamine and noradrenaline innervation and apathy, in the bilateral ventral striatum in resting-state analysis (Isella et al., 2002).

### Subcortical vascular dementia

Subcortical vascular dementia (sVaD) relates to small-vessel disease (Remy et al., 2005) and encompasses small vessel arteriosclerosis, liphyalinosis and arteriosclerosis (Remy et al., 2005), resulting in lacunar infarct occurring in distribution of small arterioles, usually in the white matter, basal ganglia, thalamus, and pons (Roman et al., 1993; Remy et al., 2005; Jelinger, 2013). Due to the anatomical disposition, sVAD include a systemic progressive dysexecutive syndrome, and apathy (Chui, 2001; Dujardin et al., 2009; Moretti et al., 2015). Starkstein et al. (2009) observed that in patients who experienced apathy in combination with depression, more white matter hyperintensities were found in the parietal lobes compared to patients without apathy and depression or in groups with apathy or depression only [see data and comments in Kos et al. (2016)]. Neuropathological data here lacks, but can be resumed from other studies.

Guimaraes et al. (2008) adapted a pathophysiological model for apathy in AD, for sVAD: ACC and OFC (involved in goal—motivated planned action) via the basolateral amygdala and nucleus accumbens, projects to the ascending frontostriatal pathway and to the dorsolateral prefrontal cortex (PFC), fundamental for the executing correct behavior (Guimaraes et al., 2008). Damage to the ACC and OFC leads to apathy (Guimaraes et al., 2008): the same result might be derived from the damage of the connecting subcortical vias, from BG to ACC and OFC (Guimaraes et al., 2008). In our opinion (Chui, 2001; Dujardin et al., 2009), we suggest a putative role for the subcortical networks in connection with the pars triangularis, the superior frontal gyrus, and the orbital operculum and may suggest that degeneration of the neural networks toward OFC and ACC can be associated to apathy, as the same result, via different pathway (Chui, 2001; Dujardin et al., 2009). Recently, white matter hyperintensities in the frontal cortical and subcortical areas have been associated with apathy (McIntosh et al., 2015), not confirmed by another study (Pluck and Brown, 2002). Behavior alterations and white-matter lacunes of basal ganglia in AD resulted in a two- to three-fold increased risk of delusions, apathy, hallucinations, and depression (Rosen et al., 2005).

Even if there are very few, and limited cases of sVAD neuroimaging dedicated study for the correlation between apathy and white matter burden, we can deduct some information in the AD patients with white matter alterations and apathy (Kos et al., 2016). In patients with high apathy, lower FA values were found within the left, right, or bilateral anterior and posterior cingulum (Ott et al., 1996; Robert et al., 2006; Kang et al., 2012; Hahn et al., 2013), with non-specific white matter changes (Tighe et al., 2012; Geda et al., 2013) and with additional increment of white matter hyper- intensities in the frontal lobes (Starkstein et al., 2009) and basal ganglia (Baggio et al., 2015), of the right thalamus and parietal regions (Kang et al., 2012). New dedicated studies should take sVAD and apathy as the principal focus of neuroimaging detection.

## Conclusive statement

Apathy is a widespread condition, which definitively increment the burden of the basal predisposing neurological conditions; there are, at the moment, not adequate clinical instrument, to evaluate it.

What do we know form animal models (Watanabe et al., 1995) is that apathy should be subserved by four different circuits, which mediates the motivational working memory, the cognitive coloring of motivation, the integration of arousal into motivation, and the reward memory into motivational response.

The information which derived from *in vivo* clinical practice do not object and refuse these circuits.

Kos et al. (2016) and Tatemichi et al. (1992) review confirms the association between abnormalities within the fronto-subcortical circuitry and apathy. In addition, this review highlights the involvement of the ACC and adds the inferior parietal cortex as a region of interest (Kos et al., 2016; Guimaraes et al., 2008; Chui, 2001; Moretti et al., 2015), relating ACC and parietal cortex in motivation and reward systems (Tekin and Cummings, 2002; Carrera and Bogousslavsky, 2006; Palmqvist et al., 2011; Rochat et al., 2013; Blundo and Gerace, 2015).

Wide neural networks support apathy: the medial frontal regions and the dorsolateral prefrontal cortex (DLPFC), the so-called “executive circuit” supports the ability to generate and maintain purposeful, goal-directed behavior (Dujardin et al., 2009; Quaranta et al., 2012). The inferior parietal cortex, described primarily by Litvan (Aarsland et al., 1999), in cortico-basal degeneration patients, where apathy is much more common than in PSP or PD [data confirmed in Moretti et al. (2005) (Hoffstaedter et al., 2013; Westerholz et al., 2014)], is involved too. By means of fMRI, the inferior parietal cortex (Westerholz et al., 2014) increased activation (among other regions in the fronto-subcortical network) during self-initiated movements and goal-directed behavior in a healthy sample (Jenkins et al., 2000; Desmurget and Sirigu, 2009; Westerholz et al., 2014; Kos et al., 2016).

Impairments in the generation of ideas for possible actions may lead to a lack of goal-directed behavior which can be associated with abnormalities in dorsolateral prefrontal areas, in caudate dorsal nucleus, and anterior thalamic nuclei (Levy and Dubois, 2006). But it has also been suggested that apathy can arise because of an inability to actually start and execute actions, which is related to the auto-activation subtype as proposed by Levy and Dubois (2006). Kos et al. (2016) suggest that this lack of volunty to move and failure to start motor programs may additionally be due to abnormalities within the inferior parietal cortex (Aarsland et al., 1999; Tekin and Cummings, 2002).

More studies should be directed toward goal specific problems;

1. Create an univocal and well accepted anatomical-pathophysiological integrated model of neural circuits involved in apathy (that should be open and should comprise apathy as a general phenomenon, and not apathy into specific pathologies, such as PD or AD)
2. Define the operative neuropsychological instruments to define in a clinical operative context apathy, in a strong predictive test, not strongly based on caregiver' referral, not limited to patient opinions, or simply by clinical observation
3. Dedicate purposed study to detect the strength of this instrument with the integrative support of modern neuroimaging techniques.

## Author contributions

RM and RS design and wrote the manuscript, analyzed the literature, and participated to the final writing and critically drafted the manuscript.

### Conflict of interest statement

The authors declare that the research was conducted in the absence of any commercial or financial relationships that could be construed as a potential conflict of interest.
