# Supplementary figures and images for: Neural Correlates for Apathy: Frontal-Prefrontal and Parietal Cortical- Subcortical Circuits
Source: Front Aging Neurosci. 2016 Dec 9;8:289. doi: 10.3389/fnagi.2016.00289 (PMC5145860; doi:10.3389/fnagi.2016.00289)

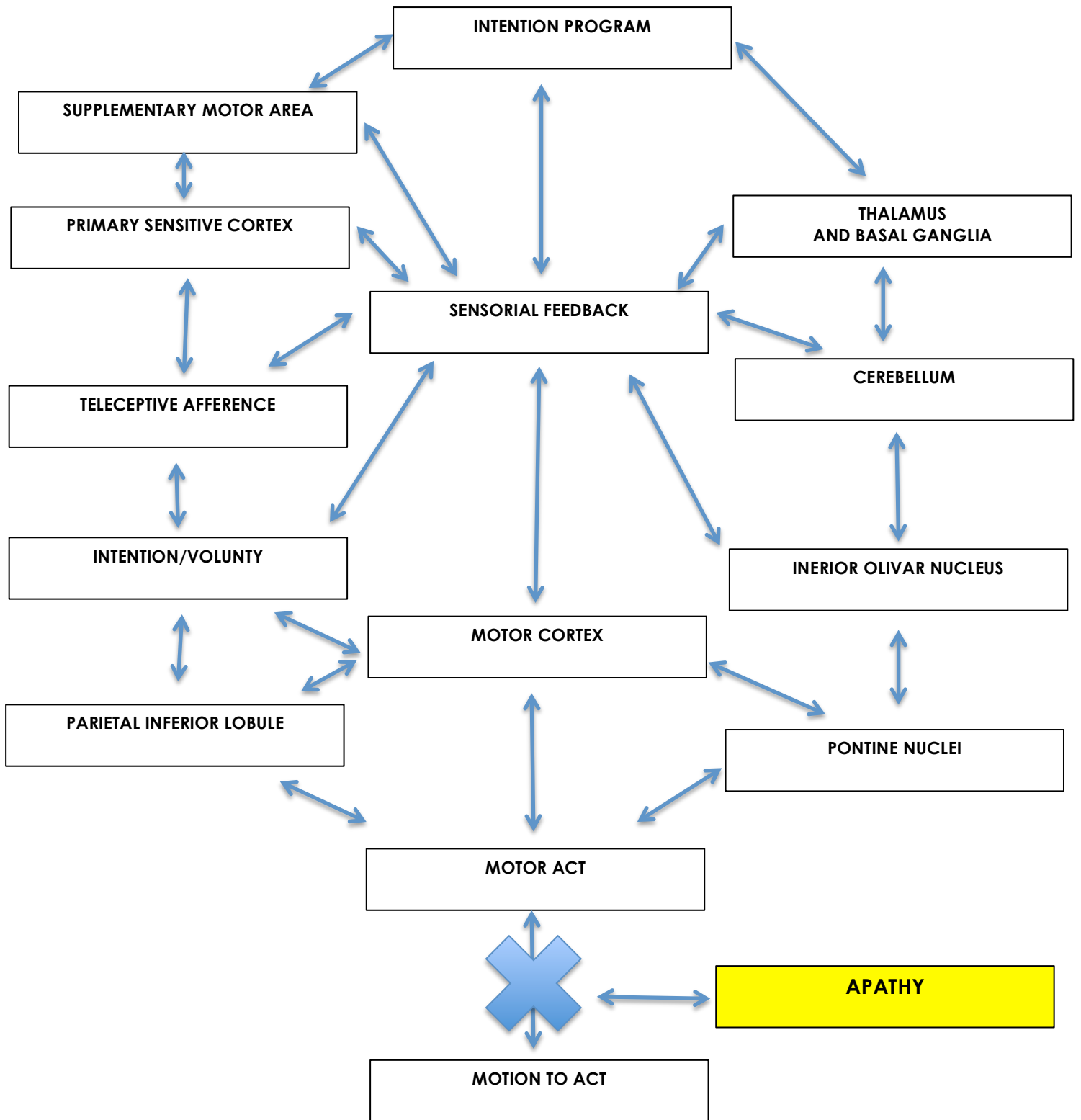

Supplement: Supplementary file 1 [file Image1.PDF]
